# Genomic characterization of multidrug-resistant *Escherichia coli* isolated from gills of *Labeo rohita:* Insight into resistome, virulence and pathogenicity

**DOI:** 10.1371/journal.pone.0351661

**Published:** 2026-06-25

**Authors:** Amit Kumar, Rahul Singh, Asma Masood, Dhouha Choukaier, Abhinav Kumar, Ankit Dilipkumar Oza, Denekew Alemayehu Alamneh

**Affiliations:** 1 Department of Zoology, School of Bioengineering and Biosciences, Lovely Professional University Phagwara, Phagwara, Punjab India; 2 Department of Basic Health Sciences in the Foundation Year for Health Colleges Program, College of Languages, Princess Nourah bint Abdulrahman University, Riyadh, Saudi Arabia; 3 Sustainable Development Research Center, Azerbaijan State Oil and Industry University, Baku, Azerbaijan; 4 Centre for Research Impact & Outcome, Chitkara University Institute of Engineering and Technology, Chitkara University, Rajpura, Punjab, India; 5 Department of Mathematical Sciences, Saveetha School of Engineering, SIMATS, Chennai, Tamilnadu, India; 6 Department of Technical Sciences, Western Caspian University, Baku, Azerbaijan; 7 Department of Mathematics, Hawassa University, Hawassa, Ethiopia; Nitte University, INDIA

## Abstract

Antibiotic resistance in aquaculture settings is an emerging issue that threatens animal and human health. In this work, a multidrug-resistant (MDR) *Escherichia coli* strain (RG3) was isolated from the gill of *Labeo rohita* obtained from a retail fish market in Punjab, India. Antimicrobial susceptibility testing revealed resistance to various antibiotic groups, with intermediate susceptibility to imipenem. Whole-genome sequencing produced a 3.70 Mb draft genome with 3,814 genes and 39 resistance determinants. Genome analysis identified a chromosomally encoded multidrug resistance phenotype, including β-lactam resistance and efflux-mediated mechanisms, together with virulence factors associated with iron acquisition, biofilm formation, and host colonization. No plasmids were predicted in the short-read assembly. Genome-based prediction suggested a high likelihood of human pathogenicity and assigned the isolate to sequence type ST8020. This study aimed to characterize an MDR *E. coli* from an aquaculture source using phenotypic and genomic approaches. The identification of a chromosomally encoded resistome together with virulence-associated traits highlights the potential of aquaculture-derived *E. coli* to act as reservoirs of antimicrobial resistance within a one health framework.

## 1. Introduction

The growing prevalence of antimicrobial resistance (AMR) is one of the biggest threats to food security, global health, and sustainable aquaculture methods. Global health, food security, and sustainable aquaculture practices are all facing significant challenges due to the growing burden of AMR. Among the many bacterial species implicated, *Escherichia coli (E. coli)* has become a prominent environmental sentinel and a leading opportunistic pathogen because of its ecological ubiquity and unmatched genomic plasticity. *E. coli* has been reported from freshwater and marine fish, including gill-associated microbiota, where its presence is considered an indicator of fecal contamination and environmental antimicrobial pressure and has been associated with opportunistic infections in fish. Some *E. coli* strains, which were once thought to be commensal with the gastrointestinal tract, have developed a broad repertoire of virulence factors and antimicrobial resistance genes that enable them to survive, adapt, and spread throughout a variety of hosts and ecological niches, including aquatic systems [1–3]. Multidrug-resistant (MDR) *E. coli* is especially concerning in the aquaculture context because of the possibility of cross-species transmission, horizontal gene transfer, and contamination of water sources used for both domestic and agricultural purposes. An important part of aquatic food systems is the freshwater fish *Labeo rohita*, which is consumed extensively in South Asia. Due to its exposure to man-made contaminants, such as antibiotics that are brought in by agricultural runoff and uncontrolled aquaculture activities, MDR bacteria can evolve and persist in a selective environment [4]. A vital interface between aquatic fauna and waterborne microbial communities, the gill microenvironment can serve as a reservoir for resistant and pathogenic microorganisms. Despite this, it is frequently disregarded.

The antibiotics oxytetracycline, florfenicol, erythromycin, sulfadiazine, and amoxicillin are extensively used in aquaculture [5] Additionally, these antibiotics play a significant role in human medicine, and their use in aquaculture may lead to the emergence of antibiotic resistance, which may be passed on to people via a number of pathways, including horizontal gene transfer. *E. coli* is a major pathogen in both community and healthcare-associated illnesses. ESBL-producing *E. coli* have spread over the world, limiting the therapeutic choices. Acquired AmpC β-lactamases in *E. coli* pose a danger to the efficacy of broad-spectrum penicillin’s and third-generation cephalosporins (3GCs) [6]. The study of AMR in bacteria isolated from aquaculture fish emphasizes the importance of looking beyond human clinical settings, supporting one Health perspective. Aquatic environments can act as reservoirs for resistant bacteria with the potential to transfer resistance genes and even virulent, pathogenic strains to humans through direct or indirect contact [7]. In *E. coli*, the transcriptional regulator CsgD plays a central role in biofilm development by activating genes responsible for curli fimbriae and cellulose synthesis, which are essential components for surface attachment and extracellular matrix formation [8]. These structures not only help bacteria adhere to surfaces but also contribute to the structural stability of biofilms. In parallel, the AcrAB-TolC efflux pump, a well-characterized multidrug resistance system, enhances antibiotic tolerance within biofilms by actively expelling a broad range of antibiotics from the bacterial cell.

The combination of WGS (whole genome sequencing) and bioinformatics, including specialised software for gene typing, resistance gene identification, and pathway mapping, provides the most in-depth understanding of MDR bacteria's genomic architecture, resistance potential, virulence, and metabolic stability. This procedure is fundamental for current microbial genomes, surveillance, epidemic analysis, and the development of new treatments and intervention techniques [9].The rapid advancement of (WGS) has revolutionized bacterial genomics, enabling comprehensive insight into the resistome, mobilome, virulome, and pan-genome of pathogenic bacteria [10]. Resources like the Comprehensive Antibiotic Resistance Database (CARD) and associated computational tools play a key role in Identifying and analysing antibiotics resistance genes [11]. PathogenFinder, VirulenceFinder [12], and Clusters of Orthologous Groups (COG) have enabled automated, high-throughput functional annotation and resistance prediction from raw genomic data. Other tools like PlasmidFinder, and MLST/CGE typing platforms provide molecular and epidemiological context to resistance evolution [13,14]. Whole-genome sequencing has revolutionized antimicrobial resistance surveillance by mapping resistomes, virulence profiles, MGEs, and gene transfer pathways. A recent study found *E. coli* strain WG5D in South Africa to harbour 49 ARGs, including those causing resistance to fluoroquinolones, tetracyclines, aminoglycosides, cephalosporins, and glycopeptides [15]. Although multidrug-resistant *E. coli* has been reported from aquaculture environments, genomic characterization of fish gill–associated isolates from India remains scarce. In particular, integrated analyses combining phenotypic resistance with whole-genome–based in silico prediction of the resistome, virulome, and pathogenic potential are limited for aquaculture-derived strains. This lack of genomic insight constrains our understanding of their ecological adaptation and public health relevance. The Present study aimed to isolate and determine the antimicrobial susceptibility profile of *E. coli* isolated from gills of *Labeo rohita,* and perform Whole Genome sequencing to identify antimicrobial resistance gene, virulence determinants and genomic characteristics and asses its potential public health relevance in the context of aquaculture-associated AMR.

## 2. Materials and methods

### 2.1. Sample collection and processing

Gill swabs were collected aseptically from 20 healthy *Labeo rohita* (rohu) from retail fish market in the Phagwara region, Punjab, India, between March and April 2025. Swabs were placed in sterile phosphate-buffered saline (PBS) to maintain bacterial viability during transport [16] and processed within 2–4 hours of collection. Samples were streaked onto Mueller–Hinton agar (MHA) plates and incubated at 37 °C for 18–24 h, aerobic condition, under standard microbiological conditions to obtain pure colonies.

### 2.2. Antibiotic susceptibility testing

The isolates were tested for antibiotic resistance by Kirby–Bauer disc diffusion on MHA following Clinical and Laboratory Standards Institute (CLSI, 2023) guidelines. Commercial antibiotic discs and antibiotic/inhibitor mixtures (amoxicillin–clavulanic acid (20/10 µg; AMC 30), erythromycin (15 µg), tetracycline (30 µg), cefotaxime (30 µg), imipenem (10 µg), ciprofloxacin (5 µg), chloramphenicol (30 µg), and gentamicin (10 µg) (HiMedia, Mumbai, India) were utilized. Lawns of bacteria were developed by uniformly inoculating cultures on MHA plates and disc placement subsequently on the agar surface. These Plates were incubated at 37 °C for 18–48 h and zone of inhibition were observed to assess the susceptibility profile [17]

### 2.3. Multiple Antibiotic Resistance Index (MARI) calculation

Multiple Antibiotic Resistance Index (MARI) for every isolate was calculated using the formula MARI = a/b,where a represents the number of antibiotics to which the isolate showed resistance and b represent the total number of antibiotics tested.isolates with MARI ≥ 0.25 were deemed to have come from sources of high-risk contamination [18].

### 2.4. Biofilm formation assay

The Congo Red Agar (CRA) method of biofilm detection on Brain Heart Infusion (BHI) agar containing Sucrose and Congo red [19]. Bacterial suspensions were adjusted to a 0.5 McFarland standard and streaked onto the plates, followed by aerobic incubation at 37 °C for 24–48 h. Reactive colonies with crystalline dry morphology were detected, while poor or non-biofilm-producers were pink. A biofilm-forming *Escherichia coli* isolate was used as a positive control, and *E. coli* MTCC 443 served as a negative control.

### 2.5. *16S rRNA* gene sequencing

Bacterial isolate RG3 was subjected to *16S rRNA* gene sequencing from, Genexplore, India. using primer set 27F(5’-AGAGTTTGATCMTGGCTCAG-3’) & 1391R (5’-GACGGGCGGTGTGTRCA-3’) primers [20]. The *obtained* sequence used to carry out BLAST analysis with the NCBI GenBank database [21]. The top ten sequences, identified based on maximum identity scores, were aligned using multiple sequence alignment tools. The Neighbour-Joining approach [22] was used to reconstruct the evolutionary history. The bootstrap consensus tree estimated from 500 replicates [23] is used to illustrate the taxon's evolutionary history. Branches representing partitions replicated in less than fifty percent of bootstrap replicates are collapsed. The proportion of duplicate trees in which the connected taxa grouped together in the bootstrap test (500 repetitions) is indicated next to each branch. The evolutionary distances were calculated using the Jukes-Cantor technique [24] and are expressed in base substitutions per site. This study consisted of 11 nucleotide sequences. All uncertain places were deleted for each sequence pair (pairwise deletion option). The final dataset had 954 positions. MEGA11 was used to perform evolutionary studies [25].

### 2.6. rMLST and whole-genome phylogenetic analysis

To achieve strain-level resolution, ribosomal multilocus sequence typing (rMLST) was conducted using the PubMLST database, which compares conserved ribosomal protein genes across bacterial taxa [26].

Whole-genome phylogenetic analysis was carried out using the Codon Tree pipeline in the BV-BRC platform. Conserved protein families were identified, aligned using MUSCLE, and a maximum-likelihood phylogenetic tree was constructed using RAxML to determine the evolutionary placement of the isolate among reference *Escherichia* genomes. [27–29].

### 2.7. Genomic DNA isolation

The Qiagen DNeasy UltraClean Microbial Kit (Cat. No. 12224−50) was used to isolate the material for both qualitative and quantitative analysis (2020). A 0.8% agarose gel was used to evaluate the quality of the isolated DNA. DNA fragments are separated based on size using agarose gel electrophoresis. After loading a tiny amount (5 μl) of the extracted DNA sample onto the gel, it was run for 30 minutes at 110 V. High-quality genomic DNA was suggested by the gel's single intact band. To determine the A260/280 ratio of genomic DNA (DNA purity), two microlitres of the sample were put into a BioTeK Epoch spectrophotometer.

### 2.8. Preparation of libraries for run chemistry

The Ion Xpress™ Plus Fragment Library Kit (Thermo Fisher Scientific, USA) was used to prepare the library, and the manufacturer's instructions for DNA fragmentation, purification, adaptor ligation, amplification, and quantification were followed. The Ion Library TaqMan® Quantitation Kit was used for the quantification process. Following the instructions, the AgilentTM High Sensitivity DNA Kit on the AgilentTM 2100 Bioanalyzer was used to measure the fragment size of the purified DNA as part of the quality control phase.

In accordance with the manufacturer's instructions, template preparation was carried out using the Ion ChefTM Instrument and the Ion 550TM Kit (Thermo Fisher Scientific, USA) after library preparation. The Ion GeneStudio™ S5 Plus System (Ion Torrent, Thermo Fisher Scientific, USA) was used to sequence the prepared libraries after they were loaded onto chips using the Ion 550™ Chip Kit.

### 2.9. De novo assembly & annotation

#### 2.9.1. Genome assembly.

Ion Torrent single-end reads were processed using the Ion Torrent Suite for adapter trimming and quality filtering with a Phred quality score cutoff of Q20. Reads shorter than 50 bp and low-quality or ambiguous reads were removed prior to assembly. The filtered high-quality reads were de novo assembled using SPAdes v3.13.0 [30]. Contigs shorter than 500 bp were excluded from downstream analysis. Assembly statistics were generated using QUAST [31], and genome completeness and contamination were assessed using CheckM v1.2.1. [32]. Details of tools and databases are mentioned in Table 1.

**Table 1:** Details of tools and databases utilized in the analysis.

Usage	Software/ Database	Version
**Genome Assembly**	SPAdes	3.13.0
**Assembly Assessment**	CheckM	1.2.1
**Annotation**	BV-BRC (https://www.bv-brc.org) – RAST Annotation Bakta	1.9.2
**Species Typing & Characterization**	PubMLST (https://pubmlst.org) DB: BIGSdb Pathogenwatch (https://pathogen.watch)	– 1.42.7 21.4.3
**Genome Comparison &** **Phylogeny**	Bakta BV-BRC	1.9.2 3.27.0
**Circos Plot**	GenoVi	0.4.3
**Antibiotic Resistance**	Resistance Gene Identifier DB: CARD BVBRC (DB: CARD, PATRIC)	6.0.3 3.2.8
**Virulence Factors**	Abricate (DB: VFDB) BV-BRC (DB: VFDB, Victors)	1.0.1 3.27.0
**Plasmid Identification**	PlasmidFinder v2.1 (https://cge.food.dtu.dk/services/PlasmidFinder/)	2.0.1
**Pathogen prediction**	PathogenWatch (https://pathogen.watch)	21.4.3
**Roary**	Pangenome analysis	v3.13.0

#### 2.9.2. Genome annotation.

The BV-BRC server (formerly PATRIC) and Bakta were used to annotate the assembled genomes [33,34]. Cellular functions and functional categorizations were inferred using BlastKOALA against the KEGG database. Metabolic features and pathways were determined using the KEGG Orthology system, and KO numbers assigned to each protein sequence were submitted to KEGG Mapper for metabolic reconstruction [35]. Circular genome representations were generated using GenoVi and predicted genes were classified into COG functional categories using the COG classifier [36].

### 2.10. Identification of genes related to antibiotic resistance, virulence factors, pathogenicity

Antimicrobial resistance genes were identified using the Resistance Gene Identifier (CARD) and BV-BRC pipelines. Virulence factors were predicted using Abricate with VFDB and the BV-BRC pipeline. PlasmidFinder identified plasmid-associated sequences (coverage thresholds: ≥ 95% identity and ≥60% coverage). Pathogenic potential was predicted using PathogenFinder and PathogenWatch tools.

### 2.11. Pangenome study

Roary pangenome program (v3.13.0)were used to analyze the pangenome of bacterial genomes 504100442-RG3 with other reference strain *E. coli*_K12_MG1655 *Escherichia coli*, and *Escherichia coli*_ER2796. [37] The GFF3 files for each genome that were used in this investigation were annotated. Roary first grouped homologous genes across the genomes using BLASTp, and the clustering results were recorded in the clustered_proteins file. Genes found in almost all genomes (≥99%) were considered the core genome while those present in only some genomes were treated as accessory genes. To visualize gene-level differences, we used the presence/absence information of the accessory genes to generate a heatmap. This clearly shows which genes are shared across the four strains and which are unique to specific isolates providing a straightforward view of their genomic similarities and differences.

## 3. Results and discussion

Of the 18 Gram-negative isolates recovered, four (RG3, RG4, RG6, and RG10) exhibited elevated MARI values (≥0.375). Among these, RG3 showed the highest MARI value (0.50) and was resistant to amoxicillin–clavulanic acid, erythromycin, cefotaxime, and tetracycline, with intermediate susceptibility to imipenem (S1 Table in S1 File). Owing to its pronounced multidrug-resistant phenotype and highest MARI score. Multidrug resistant isolate RG3, that later confirmed as *E.coli* isolated from the gill of *Labeo rohita* (Rohu), underwent whole-genome sequencing (WGS) using the Ion Torrent S5 Plus platform. The sequencing produced 5,567,180,451 bases across 29.6 million reads with an average read length of 188 bp. Quality filtering showed that 4.58 billion bases exceeded the Q20 threshold, ensuring high-quality data suitable for assembly and annotation. Assembly using SPAdes v3.13.0 resulted in a draft genome of 3.70 Mb composed of 76 contigs, with a GC content of 50.66%. The N50 value was 119,319 bp, and the largest contig measured 329,216 bp. Genome completeness, assessed by CheckM, was 77.9%, with coarse and fine consistencies of 90.5% and 88.3%, respectively. These metrics suggest a high-confidence assembly suitable for downstream genomic analyses [30,32] (Table 2).

**Table 2:** Results of genome assembly and annotation of *E. coli* RGIII.

Category	Parameter	Value
**Genome Assembly**	Raw Reads Count	5,567,180,451
	Assembly Software	SPAdes v3.13.0
	Genome Size	3,697,655 (3.6 MB)
	GC Content	50.66%
	Number of Contigs (≥ 500 bp)	76
	Largest Contig	329,216 bp
	Contig N50	119,319 bp
	Contig L50	10
	Number of Ns	0
**Genome Quality**	Coarse Consistency	90.5%
	Fine Consistency	88.3%
	CheckM Completeness	77.9%
	Genome Quality	Good
**Annotation Summary**	Total Genes	3,814
	Protein Coding Genes (CDS)	3,809
	RNA Genes	5
	Ribosomal RNAs (rRNAs)	6, 1, 1 (5S, 16S, 23S)
	Transfer RNAs (tRNAs)	62
	Repeat Regions	20
	CRISPR Arrays	2

### 3.1. Species identification and phylogenetic placement

The *16S rRNA* gene sequence of isolate RG3 showed 98.6% similarity to *E. coli* (GenBank accession NR_114042.1), and the sequence was deposited in GenBank under accession number PX096583. Consistent with this result, rMLST analysis supported its classification within the *E. coli* species based on conserved ribosomal gene profiles. Whole-genome phylogenetic reconstruction further placed RG3 within a well-supported *E. coli* cluster, grouping with *Escherichia* and closely related *Shigella* reference genomes. Collectively, these analyses confirm the taxonomic identity of RG3 as *E. coli*.

**Fig 1:**
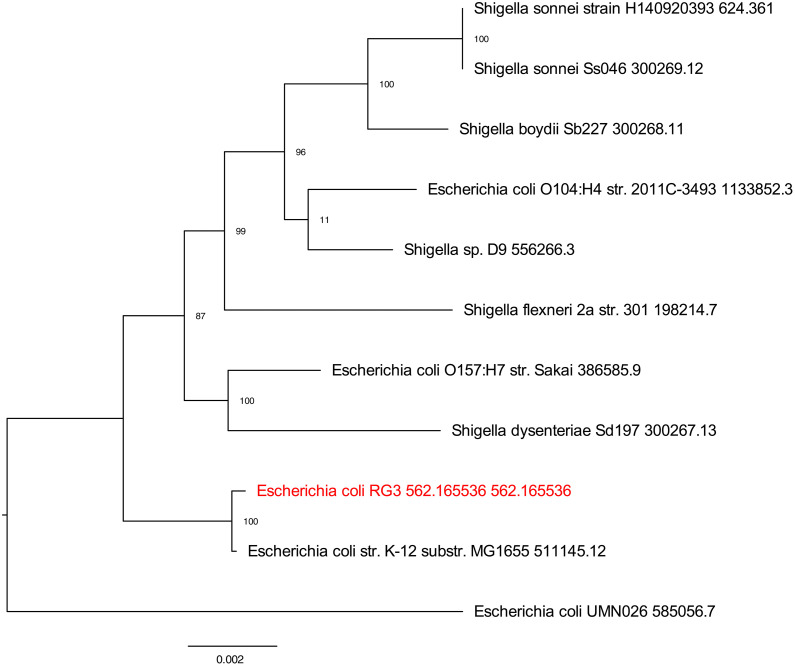
Phylogenetic trees using Codon Tree pipeline. The phylogenetic tree was constructed using conserved protein families with maximum-likelihood inference. Bootstrap values (>70%) are indicated at the nodes. The isolate RG3, which clusters within the *E. coli* clade, is distinctly separated from closely related Shigella reference genomes, confirming its taxonomic placement.

The isolate was initially identified through *16S rRNA* gene sequencing and BLASTn search, showing 98.6% identity to *Escherichia coli* (GenBank Accession: NR_114042.1). The obtained sequence was submitted to Gene bank database with accession number Escherichia PX096583.Phylogenetic reconstruction using the Neighbor-Joining algorithm in MEGA11 further confirmed its placement within the *E. coli* clade [25]. To refine strain-level classification, ribosomal multilocus sequence typing (rMLST) was conducted via PubMLST.org, which identified 40 out of 53 conserved ribosomal protein loci matching *E. coli*, validating the species identity through core gene profiling [26]. Finally, whole-genome phylogenetic analysis using the Codon Tree pipeline available on the BV-BRC platform employed conserved PATRIC Global Protein Families (PGFams), with multiple sequence alignments generated via MUSCLE and maximum-likelihood inference through RAxML, placing the isolate in a well-supported clade of *E. coli* and *Shigella* reference genomes [27–29]. This integrative approach confirmed the taxonomic identity and evolutionary placement of the isolate RG3 as *E. coli*. (Fig 1)

### 3.2. Geno Vi circular map of genome

**Fig 2:**
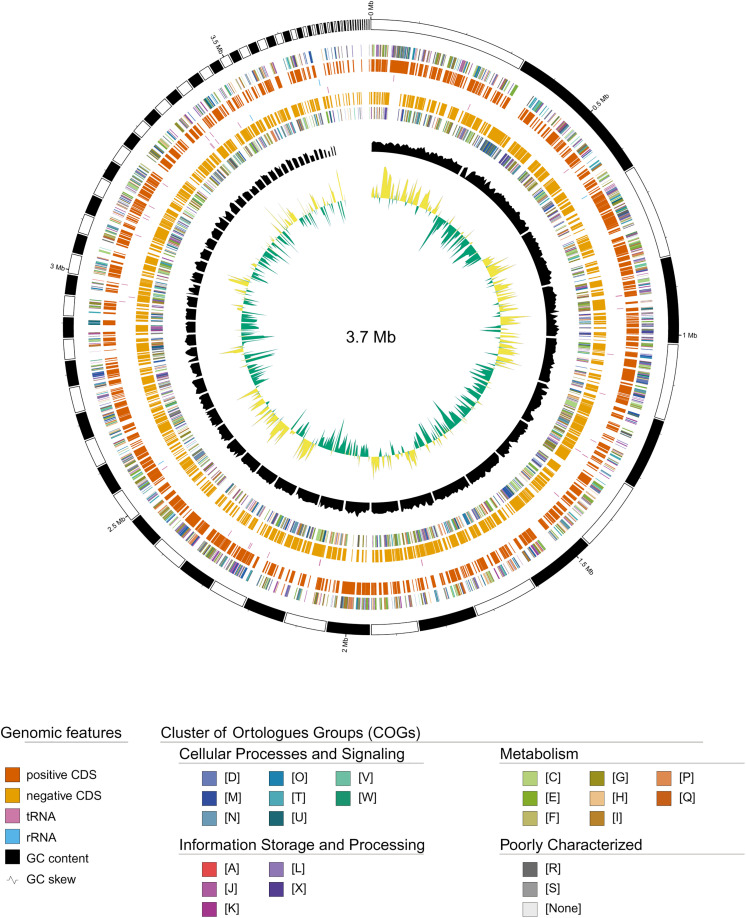
GenoVi circular map of the draft genome of *E. coli* RG3 (-s draft -cs autumn). Each contig is represented as separated bands of one circular representation. Labelling from outside to the inside: Contigs; COGs on the forward strand; CDS, tRNAs, and rRNAs on the forward strand; CDS, tRNAs, and rRNAs on the reverse strand; COGs on the reverse strand; GC content; GC skew.

The circular genome map (Fig 2) shows the distribution of coding sequences on both strands along with tRNAs and rRNAs across the 3.7 Mb draft genome. Variations in GC content and GC skew were observed throughout the genome, indicating regions of differential nucleotide composition and potential replication-associated asymmetry. Functional annotation based on COG categories demonstrates a predominance of genes associated with metabolism, information storage, and cellular processes, reflecting the metabolic versatility and adaptive potential of the isolate**.**

### 3.3. KEGG pathway annotation using BV-BRC

**Fig 3:**
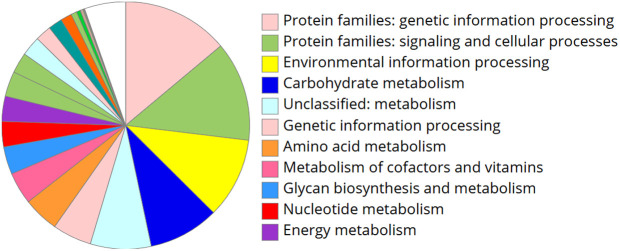
KEGG Pathway Annotation.

BV-BRC (RAST) provides a reliable and precise annotation of the genome design and execution differ from those of BlastKoala (Kegg). As a result, the genome's predicted protein sequences were used to conduct this functional annotation. BlastKoala categorised 920 KEGG numbers (68.8%) from 2519 entries (protein sequences) to 20 functional groups. Based upon this annotation most common metabolic route was that of protein families, and the major protein family identified was linked to genetic information processing. (**Fig 3**). Through KEGG Mapper, the data were organized into 234 biological functions, 37 categories of genes and proteins, and 59 metabolic pathway modules. The distribution of genes assigned to different pathways is shown in Table 3.

**Table 3:** KEGG Pathway distribution.

Level-I	Level-II	Gene Counts
09100 Metabolism	Carbohydrate metabolism	332
	Amino acid metabolism	217
	Metabolism of cofactors and vitamins	152
	Energy metabolism	138
	Nucleotide metabolism	94
	Glycan biosynthesis and metabolism	132
	Lipid metabolism	68
	Metabolism of other amino acids	62
	Metabolism of terpenoids and polyketides	28
	Biosynthesis of other secondary metabolites	43
	Xenobiotics biodegradation and metabolism	58
0091Genetic information and processing	Transcription	2
	Translation	63
	Folding, sorting and degradation	33
	Replication and error	61
A09130 Environment Information Processing	Membrane transport	191
	Signal transduction	151
09140 Cellular Processes	Transport and catabolism	6
	Cell growth and death	14
	Cellular community -prokaryotes	118
	Cell motility	48

The KEGG pathway annotation bacterial genome reveals a comprehensive functional landscape that highlights both metabolic versatility and adaptive capacity. With 68.8% of the predicted protein sequences assigned to 920 KEGG Orthology (KO) numbers across 20 functional groups, this analysis offers a broad view of the organism’s biological potential. Most of the genes in this genome fall, unsurprisingly, into the big metabolic categories. Carbohydrate metabolism is the largest group, with about 332 genes, and amino acid metabolism comes next with 217. These two sets together explain how the bacterium generates energy and builds biomass, basically helping it make the best use of sugars and amino acids when nutrients are abundant [35,38].

The Another notable cluster is cofactor and vitamin metabolism (152 genes), along with energy metabolism (138 genes). These aren’t just “extras”—they are central to keeping metabolic reactions moving and making sure energy production doesn’t stall. When it comes to genetic information processing, the numbers are again fairly high. Translation alone accounts for 63 genes. Protein folding, sorting, and degradation bring in another 33, while replication and repair add 61 more. This suggests the bacterium can maintain its genetic stability and fix problems when they arise. Only two genes were assigned to the transcription-related category in the KEGG functional classification. This likely reflects limitations of pathway mapping or database annotation rather than a true reduction in transcriptional capacity; therefore, no biological inference was drawn from this observation. Adaptability to the environment is clear from the presence of 151 genes in signal transduction and 191 in membrane transport. These give the cell a way to sense conditions around it and adjust. That sort of flexibility is important in variable or competitive environments [39]. Alongside this, genes connected with community behaviour (118) and motility [48] point toward traits like biofilm formation, colonization, and active movement.

Finally, the presence of genes tied to growth, death, transport, and catabolism hints at how the genome balances regulation and metabolism to keep populations stable and recycle nutrients.. Metabolic flexibility, indicated by the dominance of genes related to carbohydrate and amino acid metabolism, is crucial for survival in variable aquatic environments like fish gills. Enhanced membrane transport and signal transduction pathways allow for better sensing of environmental stressors, including antimicrobial substances. Additionally, pathways for xenobiotic degradation and cofactor metabolism may enhance tolerance to antibiotics and contaminants in aquaculture. These features suggest an adaptive lifestyle focused on persistence amid fluctuating environmental pressures.

### 3.4. COG (Cluster of orthologous) functional classifications of predicted proteins

**Fig 4:**
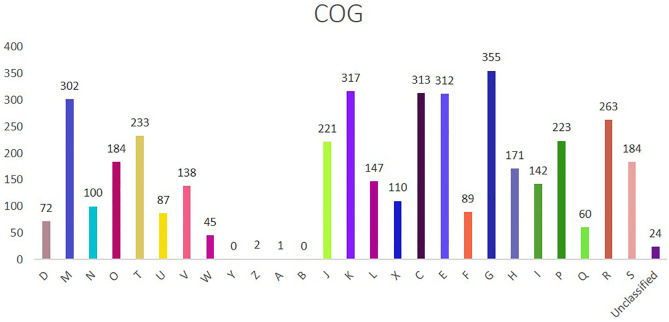
COG functional classification of predicted proteins. **A**-RNA processing and modification, **B**-Chromatin structure and dynamics, **C**-Energy production and conversion, **D**-Cell cycle control, cell division, chromosome partitioning, **E**-Amino acid transport and metabolism, **F**-Nucleotide transport and metabolism, **G**-Carbohydrate transport and metabolism, **H**-Coenzyme transport and metabolism, **I**-Lipid transport and metabolism, **J**-Translation, ribosomal structure and biogenesis, **K**-Transcription, **L**: Replication, recombination and repair, **M**-Cell wall/membrane/envelope biogenesis, **N**- Cell motility, O-Posttranslational modification, protein turnover, chaperones, **P**-Inorganic ion transport and metabolism, **Q**-Secondary metabolites biosynthesis, transport and catabolism, **R**-General function prediction only, **S**: Function unknown, **T**-Signal transduction mechanisms, **U**-Intracellular trafficking, secretion, and vesicular transport, **V**-Defence mechanisms, **W**-Extracellular structures, **X**-Mobilome: prophages, transposons, **Y**-Nuclear structure, **Z**-Cytoskeleton.

COG functional analysis (Fig 4) showed a high proportion of genes involved in carbohydrate transport and metabolism, transcription, and energy production, indicating strong metabolic flexibility and regulatory capacity required for survival in fluctuating aquatic environments. Of the 3,814 projected coding sequences, 3,662 (95.99%) were mapped to COG functional categories using the Genovi pipeline. The most frequent class contained the highest number of genes, class G (carbohydrate transport and metabolism; 355), followed by transcription (K; 317), C (energy production and conversion; 313), and E (amino acid transport and metabolism; 312), reflecting a metabolically diverse genome. Transport and structural function classes like M (cell wall/membrane/envelope biogenesis; 302), T (signal transduction; 233), and P (inorganic ion transport; 223), J (translation; 221), also were well represented and allowed adaptation to different environments. Replication (L; 147), and defence mechanisms (V; 138) underscore genome plasticity and environmental responsiveness as well. Low B coverage (chromatin structure; 1) and W coverage (extracellular structures; 45) is consistent with typical prokaryotic genomes.

The genomic fingerprint of the isolate illustrates high metabolic fitness, nutrient flexibility, and protein synthesis ability necessary characteristics to thrive in complex environments like aquaculture systems [36]. Augmentation of function categories V (Defence mechanisms, 138 genes) and M (Cell wall/membrane/envelope biogenesis, 302 genes) marks increased resistance capability and structural flexibility. In addition, the T class (Signal transduction mechanisms, 233 genes) high rank suggests a sophisticated capacity for sensory perception and response to environmental signals. Impressive as it is, the vast majority of genes fall into the S (Function unknown, 184 genes) and unclassified (24 genes) categories, which suggest the occurrence of potentially novel, strain-specific elements that are worthy of functional exploration.

### 3.5. Genomic characterization of antimicrobial resistance determinants

Comprehensive resistome analysis using the Resistance Gene Identifier (RGI) v6.0.3, leveraging curated homology and SNP models from the CARD (Comprehensive antibiotic resistance database) 2023 [40], identified 39 high-confidence AMR genes in the *E. coli* RG3 genome (S2 Table in S1 File). The detected genes encompassed a broad spectrum of antibiotic classes, including β-lactams, polymyxins, aminoglycosides, tetracyclines, fluoroquinolones, and macrolides, reflecting a robust multidrug resistance (MDR) profile.

The antibiotic resistance gene (ARG) profile of the bacterial genome detected by CARD-RGI is a detailed illustration of the complex mechanism’s bacteria employ to resist antimicrobial drugs. Efflux pumps dominate the resistome most significantly, with yojI, acrD, emrB, emrA, and TolC mediating resistance through the active efflux of a broad range of antibiotics including aminoglycosides, fluoroquinolones, macrolides, tetracyclines, and β-lactams. This widespread dependence on efflux systems is a indicator for multidrug resistance among Gram-negative bacteria, lowering intracellular drug concentrations to sublethal and favouring survival under antibiotic stress [41,42]. Target modification genes such as PmrF, eptA, bacA, and Ugd play a central role in resistance by modifying bacterial envelope motifs, decreasing antibiotic binding specificity, specifically against polymyxins—drugs that are usually kept as last resort. They show how bacteria reorganize their outer membranes to counteract the effectiveness of antibiotics, well-documented in clinical multidrug-resistant pathogens [43]. Mutational resistotypes were also predicted from gene variants such as soxR, soxS, and acrR, which using CARD's SNP model.MarA, soxR, and soxS regulatory gene mutations underscore the relevance of global transcriptional control in coordinating resistance, not only modulating efflux pump expression but also membrane permeability and target protection. These regulatory networks operate in synergy and have been commonly termed as being significant modulators of *Escherichia coli* multidrug resistance phenotypes and other affiliated bacteria [44,45].

The presence of the *ampC* β-lactamase gene confirms enzymatic inactivation as another resistance mode, hydrolysing β-lactam antibiotics to neutralize their bactericidal activity—a prolific mechanism in many Gram-negative bacteria that poses serious treatment challenges [46]. Additionally, *ampC* β-lactamase was detected with 100% identity, confirming the potential for an ampC β-lactamase phenotype. This genomic finding aligned with resistance to cefotaxime.No plasmids were predicted by PlasmidFinder in the short-read assembly; however, short-read sequencing may limit the resolution of extrachromosomal elements, and long-read approaches would provide definitive structural characterization.. Collectively, this resistome underscores the bacterium’s robust and versatile defence toolkit against a spectrum of antibiotics, reflecting evolutionary adaptations to antimicrobial pressure commonly seen in environmental and clinical isolates alike. This characterization informs future efforts in developing targeted therapies and guiding antibiotic stewardship

### 3.6. Pathogen finder

Comparison with Pathogenwatch indicates that the *E. coli* isolate being studied in this work has a high potential of infecting humans. High confidence to the pathogenic grouping is provided by the high value of probability of prediction (0.925) that was achieved with a stringent 100% identity cut-off. The strategy of using only exact genetic equivalents reduces uncertainty and ensure correctness of the prediction [47]. Of 521 genetic matches in the genome, 502 matched gene families which were already linked with pathogenicity and 19 with non-pathogenic families only. Though the coverage of the genome was quite small at 14.44%, the number of virulence-associated genes shows a humongous potential for infection (Table 4).

**Table 4:** Pathogen Finder prediction metrics for *E. coli* RG3.

Min Identity Threshold	100.0
Z-THRESHOLD	25.37
Prediction Score	2571.428
Probability of being human pathogen	0.925
Matches	521
Genome Coverage (%)	14.44
Pathogenic Families Matched	502
Non-Pathogenic Families Matched	19
The organism is predicted as human pathogenic	YES

In addition, the high prediction score (2,571.428) and Z-threshold value (25.37) confirm the power and evidence of such results in that it is very unlikely to explain such results in terms of chance alone. Such categorical dominance by pathogenic gene content agrees with earlier studies revealing that human-infective *E. coli* strains tend to be found with a certain set of virulence and resistance genes (Cosentino et al., 2013). Based on such genetic origin, the isolate is deserving of closer observation and a full analysis including antimicrobial susceptibility and molecular epidemiology, especially when cultured in clinical or environmental specimens. Pathogenicity was inferred from genome-based prediction tools, and no in vivo infection assays were performed; therefore, these findings indicate potential rather than confirmed virulence.

### 3.7. Virulence gene landscape

Virulence gene analysis using VFDB and Victors databases identified 24 virulence factors from VFDB and 131 additional factors from Victors (S3 Table in S1 File). Key virulence determinants included siderophore biosynthesis genes (*entABCDEF*, *fepABCDG*), curli-associated biofilm genes (*csgD*, *csgF*, *csgG*), and outer membrane proteins (*ompA*) The detection of csgD, a master regulator of biofilm pathways, together with structural genes csgF and csgG, confirms the genomic potential of your *E. coli RG3* strain to form biofilms [8].This is strongly aligned with the Congo red assay result. This concordance between phenotypic biofilm formation and the presence of curli-associated *csg* genes demonstrates a clear genotype–phenotype correlation, reinforcing the functional validity of the genomic prediction.

The presence of multiple secretion and iron acquisition genes suggests an enhanced ability to colonize and survive within host tissues. These features indicate that RG3 is capable of biofilm formation, epithelial adhesion, and iron scavenging, enhancing its virulence potential. In aquaculture environments, such capabilities can facilitate long-term persistence and contribute to pathogenicity in both fish and humans. The microbial community associated with fish gills more closely mirrors that of the surrounding water than the gut microbiota, due to continuous environmental exposure that enables microbial exchange and colonization by diverse, potentially pathogenic bacteria [48].

The genomic analysis of *E. coli* RG3 revealed a multidrug-resistant phenotype characterized by a diverse chromosomally encoded resistome, primarily consisting of efflux systems and ampC β-lactamase. The identification of biofilm-associated genes, siderophore systems, and adhesion-related factors suggests a capacity for host colonization and persistence. Pathogen Finder predictions corroborated its designation as a possible human pathogen. Collectively, these attributes underscore the clinical and ecological significance of this aquaculture-related strain.

### 3.8. Comparative genomic analysis of *E. coli* strain RG3

**Fig 5:**
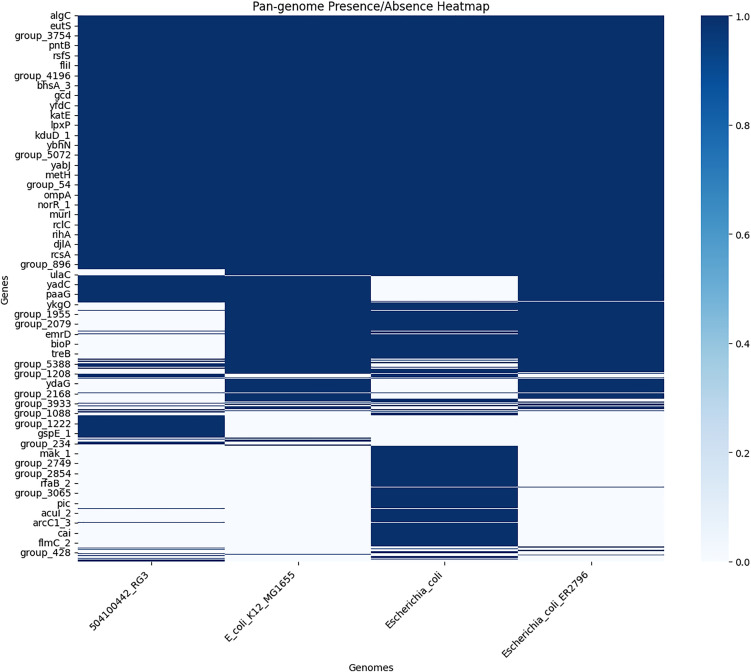
The presence/absence heatmap.

A comparative genomic analysis was performed between RG3 and three selected *E. coli* reference genomes to examine shared and strain-specific gene content. The presence/absence heatmap (Fig 5) clearly indicate that RG3 shares a substantial proportion of its gene repertoire with the reference genomes, highlighting a conserved core genome. Notably, the heatmap shows strong conservation across genes involved in essential metabolic processes (e.g., algC, eutS, rsfS, murI, ompA), which are consistently present in all strains analyzed. This supports the idea that *E. coli* maintains a highly conserved backbone necessary for fundamental cellular functions [49,50]. While based on a limited set of reference genomes, this comparison provides a useful preliminary overview of conserved and variable genomic features of RG3.

**Fig 6:**
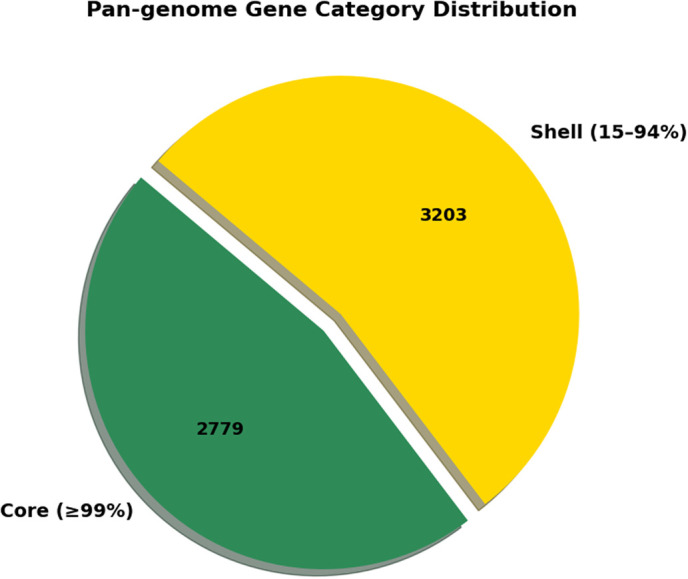
Pan genome gene category distribution.

The pan-genome gene category distribution Fig 6 further illustrates the balance between genetic conservation and diversity. A total of 2,779 genes (≥99%) were identified as part of the core genome, reflecting essential housekeeping functions and evolutionary stability across *E. coli* strains. Meanwhile, 3,203 genes (15–94%) were categorized as shell genes, which represent accessory functions contributing to strain-specific adaptation, environmental survival, and pathogenic potential. The relatively large proportion of shell genes indicates that RG3 harbours a flexible genome structure, in line with the open nature of the *E. coli* pan-genome, where continuous gene acquisition expands the repertoire of adaptive traits [51,52]. (Fig 6).

Importantly, the distribution of shell genes in RG3 highlights potential roles in environmental resilience and virulence, a finding consistent with previous reports that adaptive gene content drives niche specialization and pathogenicity in *E. coli* [53]. Given that RG3 was isolated from the gills of *Labeo rohita,* the enrichment of shell genes may contribute to its survival in aquatic environments, where fluctuating nutrient availability and antimicrobial pressures are prevalent.

Collectively, these results suggest that while RG3 retains a conserved genomic core typical of *E. coli*, it also possesses a significant accessory genome fraction, which may underpin its ecological fitness, antimicrobial resistance, and pathogenic potential in aquatic ecosystems.

It demonstrates that RG3 shares a conserved core gene set with reference *E. coli* genomes while also possessing strain-specific accessory genes. This pattern supports the open nature of the *E. coli* pan-genome and suggests genomic plasticity associated with environmental adaptation.

## 4. Conclusion

Whole-genome sequencing (WGS) *E. coli* strain RG3, recovered from the gill of *Labeo rohita*, revealed a multidrug-resistant and virulent genomic profile, even in the absence of plasmids. The presence of chromosomally encoded resistance genes, along with virulence factors linked to host colonization and immune evasion, reflects the adaptive potential of this aquatic isolate. Functional genome annotation also highlighted the strain’s metabolic flexibility and resilience in its environment. These findings highlight the value of WGS and software as a powerful tool for detailed detection of resistance genes, virulence traits, and human pathogenic potential in bacteria from non-clinical environments. Although MDR bacteria in aquaculture are a growing concern for public health, genomic data on such strains especially from India remain limited. This study underscores the urgent need to strengthen WGS-based surveillance of resistant bacteria in aquaculture, in line with the One Health approach that connects human, animal, and environmental health.

### 4.1. Future prospective

This study acknowledges some limitations: its genomic analysis relied on a single draft genome and a limited set of reference strains, potentially overlooking the full diversity of aquaculture-associated *E. coli.* Retail sample origins restricted access to important farm-level metadata, including antibiotic usage and water quality. Plasmid detection was based on short-read sequencing, hindering confirmation of plasmid architecture and genomic context of resistance genes. Virulence potential assessments were made using genomic prediction tools without in vivo validation. Despite a draft genome completeness of 77.9%, which may limit accessory element resolution, however, it remained sufficient for reliable resistome, virulence, and functional genomic characterization.

Future investigations involving a larger number of samples and isolates for comparative genomic and pan-genome analyses, together with improved assembly continuity will provide deeper insights into the resistome, mobilome and transmission dynamics of aquaculture-associated *E. coli*.

**Accession number of SRA data**-PRJNA1310943

## Supporting information

S1 File S1 Dataset. Gene presence–absence matrix generated by Roary. **S1 Table.** Antimicrobial Susceptibility Profile of *Escherichia coli* RG3. **S2 Table**. Antimicrobial resistance genes identified in *E. coli* RG3 using the CARD-RGI pipeline. **S3 Table**. Virulence factors identified in E. coli RG3 using the BV-BRC Victors database.
(RAR)
